# Spontaneous Hepatobiliary Disease in a Breeding Colony of *Aotus* spp.

**DOI:** 10.1111/jmp.70114

**Published:** 2026-09-10

**Authors:** M. E. Hensel, G. W. Wilkerson, L. Yan, C. L. Hodo, S. M. Kezar

**Affiliations:** ^1^ Michale E. Keeling Center for Comparative Medicine and Research The University of Texas MD Anderson Cancer Center Bastrop Texas USA; ^2^ Division of Comparative Medicine, School of Medicine The University of North Carolina at Chapel Hill Chapel Hill North Carolina USA

**Keywords:** cholangiocarcinoma, cholecystitis, hepatopathy, liver disease, owl monkey

## Abstract

**Background:**

Owl monkeys are nonhuman primate species in the genus *Aotus*. The Michale E. Keeling Center for Comparative Medicine and Research maintains an Owl Monkey Breeding and Research Resource composed of four species of owl monkeys: *Aotus nancymai* , *A. vociferans* , *A. azarae* , and *A. griseimembra.* Disease of the hepatobiliary system has not been well characterized in *Aotus.*

**Methods:**

Necropsy records from a 17‐year period were reviewed to characterize the gross and microscopic lesions of liver disease.

**Results:**

316 adult owl monkeys were submitted for necropsy in the review period; 22 of 316 (6.96%) were diagnosed with hepatobiliary disease. Cardiac‐associated hepatopathy was the most common diagnosis (6/22). Previously unreported conditions included cystic mucinous hyperplasia (1/22), acute cholangiohepatitis (1/22), idiopathic end‐stage hepatopathy (1/22), cholangiocarcinoma (2/22), and acute cholecystitis (4/22). Vitamin E deficiency anemia (3/22) and hemosiderosis (4/22) have been reported in the genus, and these diagnoses were noted.

**Conclusions:**

The spectrum of hepatobiliary pathology in owl monkeys is broader than previous reports have suggested.

## 1 Introduction

Owl monkeys (*Aotus* spp.) are a nocturnal haplorrhine nonhuman primate species native to countries of Central and South America. *Aotus* spp., including *Aotus nancymai* , *Aotus azarae* , *Aotus griseimembra* , and *Aotus vociferans* , are also bred and used for biomedical research, especially malaria investigations [1, 2, 3]. Information about the hepatobiliary tract and its impact on animal health has not been as thoroughly investigated as disease of the cardiovascular and renal systems. However, this gap in the literature has been the subject of recent prospective analyses of hepatic function and ultrasonographic findings in *Aotus azarae infulatus* and a retrospective analysis of obstructive hepatopathy from biliary disease in our research population [4, 5, 6].

From reviews of mortality in research populations of *Aotus* spp. and individual case reports the following conditions have been reported: hepatic lipidosis, acute toxic hepatitis of undefined etiology, cholesterol cholelithiasis, presumptive hemochromatosis, hypoxia‐associated centrilobular coagulative necrosis from vitamin E deficiency, obstructive hepatopathy associated with cholelithiasis, and single reports of spontaneous adenocarcinoma of the gallbladder and a gallbladder hematoma [4, 6, 7, 8, 9, 10, 11, 12].

Herein we present an overview of spontaneous hepatobiliary pathology in owl monkeys that resulted in clinical signs of hepatic disease or lesions at necropsy. Clinical disease was evaluated by serum chemistry and physical exam results. Gross and histologic evaluation were used to characterize hepatic lesions.

## 2 Materials and Methods

### 2.1 Animals and Tissue Sampling

The Michale E. Keeling Center for Comparative Medicine and Research houses four species of owl monkeys including *Aotus nancymai* (AN), *A. vociferans* (AV), *A. azarae* (AA), and *A. griseimembra* (AG). Procedures involving animals were conducted in compliance with applicable regulations including oversight by the University of Texas MD Anderson Cancer Center Institutional Animal Care and Use Committee (protocol # 0329‐RNXX). Animal care and husbandry conformed to practices established by the AAALACi, The Guide for the Care and Use of Laboratory Animals, and the United States Department of Agriculture Animal Welfare Act and Animal Welfare Regulations [13]. All animals were experimentally naïve and were part of the Owl Monkey Breeding and Research Resource.

Animals were housed in compatible social groups composed of a breeding pair and recent offspring unless health status necessitated individual housing for supportive care. Animal housing rooms were maintained at 75°F–80°F with a relative humidity between 50%–70% and at least 10 complete air changes per hour. A 12:12 h light dark cycle was maintained in animal rooms. Animals were housed either in 48″ D × 60.5″ H × 36″ W Britz cages or 28″ D × 31.5″ H × 32.75″ W stainless steel cages with thermoneutral sanitizeable plastic nest boxes and a variety of physical enrichment including ladders, barrels, and manipulanda such as whiffle balls, plastic chains, and plastic figurines. Caging was sanitized on a biweekly rotation with caging and nest boxes sanitized on opposite weeks to allow maintenance of olfactory cues. Animals were fed Teklad 7195, Hi‐Fiber Primate diet supplemented with daily produce items (e.g., oranges, sweet potato, peanuts, meal worms). Reverse osmosis treated water was provided ad libitum via a lixit system. Animals received supplemental injections of 300 IU Vitamin E subcutaneously every 2 months.

Necropsy reports and medical records from a 17‐year period (2008–2025) at the Keeling Center were reviewed for clinical histories (including serum chemistry and clinical signs) consistent with liver disease and necropsy reports that cited significant liver pathology. Neonates were excluded from the search as insufficient numbers were submitted to draw population level conclusions. During this period 316 adult owl monkeys were either humanely euthanized via sodium pentobarbital overdose or found deceased and submitted for a complete necropsy.

At necropsy, a standard tissue list was collected representing all major organ systems according to institutional standard operating policy in 10% neutral buffered formalin and were routinely processed, embedded, and stained with hematoxylin and eosin. Selected samples were evaluated with Perl's for iron, Chandler's reticulin to assess hepatic cord architecture, and Masson's trichrome to evaluate fibrosis. Immunohistochemistry for cytokeratin‐7 (clone RCK105; 1:500; Invitrogen) was applied to characterize the neoplastic population. Necropsy and microscopic evaluation were performed by board‐certified veterinary anatomic pathologists. In many instances, especially in aged animals, more than one diagnosis was identified as a comorbidity. For the purposes of this manuscript, the lesions were categorized based on presumed etiology that led to liver disease.

### 2.2 Microbiology Culture

A sample from the gallbladder contents of Cases 2–4 collected at the time of necropsy with aerobic swabs was inoculated on Blood Agar, MacConkey Agar, Chocolate Agar and incubated for 24 h at 36°C–38°C with CO2 (5%–10%). A sample of the gallbladder swab was also inoculated into PEA & blood agar and incubated at 36°C–38°C for anaerobic culture. Colony isolates from the different media were then screened for organism identification using API 20E (bioMerieux).

### 2.3 Chemistry and Complete Blood Count

Whole blood was collected in a plain red top tube (BD vacutainer Ref # 366688). After the blood was fully clotted, serum was separated by centrifugation at 1300 *g* for 10 min. Chemistry parameters were measured on the Beckman Coulter AU680 Chemistry Analyzer. Whole blood was collected in a K2EDTA tube (BD vacutainer Ref # 367841). Hematology parameters were measured on the Siemens Advia 120 Hematology Analyzer. Serum chemistry and hematology values were compared to in‐house reference intervals for *Aotus* species [14].

## 3 Results

From a 17‐year period (2008–2025), 316 owl monkeys were submitted for complete necropsy and histologic evaluation. Of the 316, 22 (6.96%) were diagnosed with hepatobiliary disease based on clinical signs, hematologic analysis, and confirmed by liver lesions at necropsy. Species and population information for the animals diagnosed with liver disease is listed in Table 1. Animals ranged in age from 1 year to 24 years old and included 12 AN, 2 AV, 2 AA, and 6 AG (Table 1). The most common diagnoses are listed in Table 2.

**TABLE 1:** Species and sex distribution of *Aotus* spp. with liver disease.

Species	Total study population	Liver disease	Males	Females
*A. nancymai*	255	12	9	3
*A. vociferans*	29	2	1	1
*A. azarae*	12	2	0	2
*A. griseimembra*	20	6	4	2

**TABLE 2:** Infectious, degenerative, and neoplastic conditions identified in the hepatobiliary system of *Aotus* spp.

	Diagnosis	Number affected	Species	Sex	Median age in years (range)
Cases 1–4	Ascending cholecystitis	4	AN (2) AV (1) AA (1)	M (3) F (1)	13.5 (9–15)
Case 5	Acute cholangiohepatitis	1	AA	F	17
Cases 6–8	Vitamin E deficiency	3	AG (3)	M (1) F (2)	4 (1–8)
Cases 9–14	Cardiac‐associated hepatopathy	6	AN (5) AG (1)	M (5) F (1)	15.5 (9–20)
Case 15	End‐stage disease, undetermined etiology	1	AV (1)	M (0) F (1)	10
Cases 16–19	Hemosiderosis	4	AN (2) AG (2)	M (4) F (0)	14 (8–23)
Case 20	Cystic mucinous hyperplasia of the gallbladder	1	AN (1)	F	13
Cases 21–22	Cholangiocarcinoma	2	AN (2)	M (1) F (1)	12

Abbreviations: AA, *A. azarae* ; AG, *Aotus griseimembra* ; AN, *Aotus nancymai* ; AV, *A. vociferans* ; F, female; M, male.

### 3.1 Infectious

Four owl monkeys were diagnosed with cholecystitis (Cases 1–4). The clinical signs for cholecystitis varied amongst the cases. Case 1 was a 14‐year‐old male AV euthanized due to marked weight loss, hepatomegaly, and cardiovascular compromise. Necropsy revealed cardiomegaly, moderate intimal thickening of the thoracic aorta, an enlarged liver (5.21% body weight [BW]; Table 4), and 1–3 mm dark green to black friable material in the gallbladder. Histologically, the gallbladder wall was thickened by edema and fibrosis, and the mucosa was infiltrated by neutrophils and nodules of lymphoid hyperplasia. The liver had mild lesions that consisted of foci of hepatocellular degeneration and loss with bridging fibrosis and bile duct hyperplasia.

**FIGURE 1:**
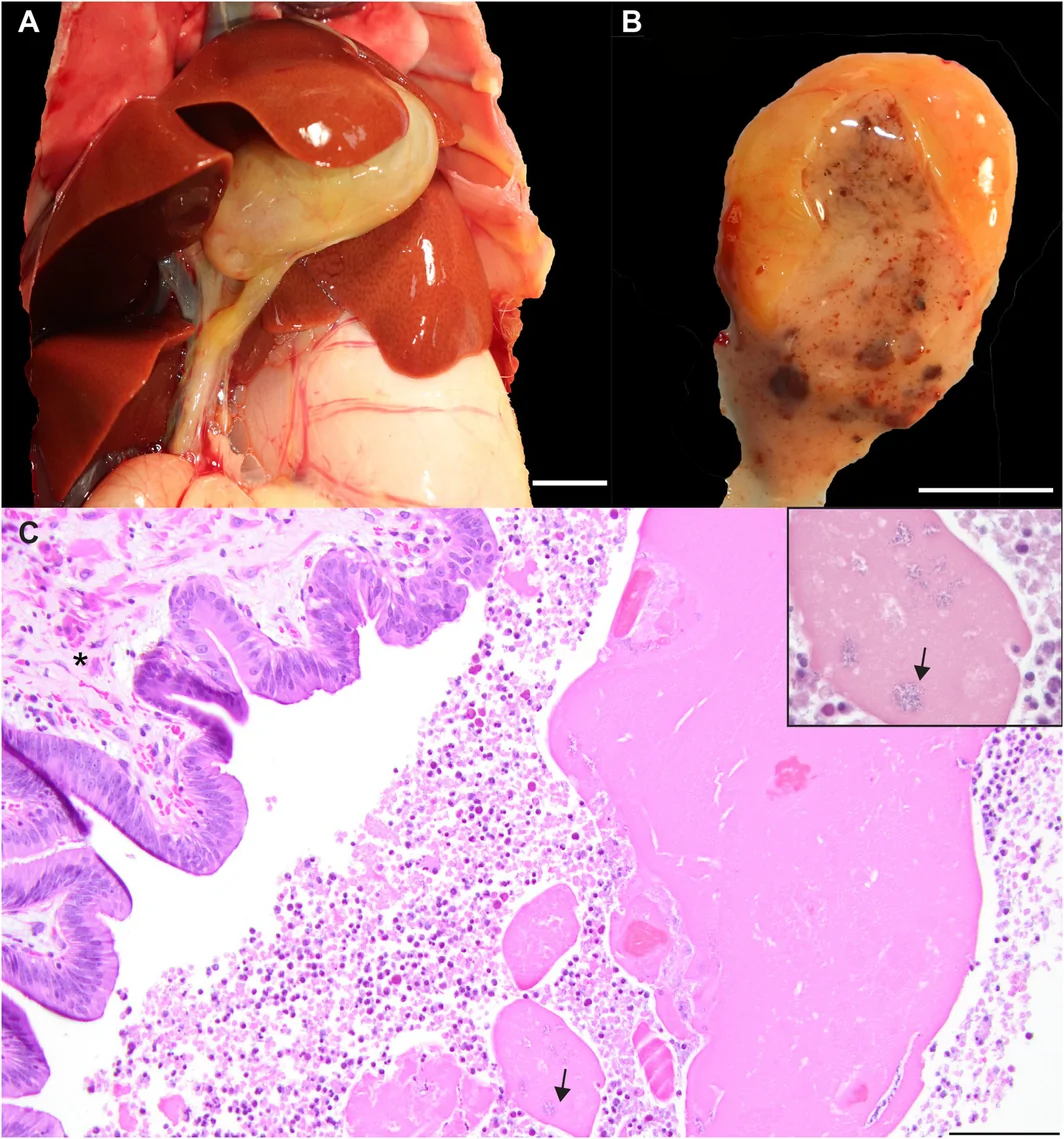
Gross and microscopic lesions of acute bacterial cholecystitis, Case 4, *Aotus nancymai* . (A) Gross image of liver and gallbladder. The gallbladder is tan‐yellow, opaque, and distended. The common bile duct is dilated and expanded by edema. Scale bar = 1 cm. (B) Gallbladder: Gross image of the gallbladder with opaque contents containing blood clots and friable choleliths. Scale bar = 1 cm. (C) Histology of the gallbladder shows mucosal hyperplasia, submucosal edema (*), and intraluminal neutrophilic inflammation with intralesional short rod‐shaped bacteria (arrow). Hematoxylin and eosin (HE), 4×. Inset, higher magnification of the bacteria (arrow). HE, 40×.

Culture results were available for Cases 2–4, which all had pure cultures of 3+ *E. coli* isolated from the gallbladder contents. Case 2 was a 9‐year‐old female AA that died spontaneously after birthing an apparently healthy infant. Over the 24‐h post‐partum period, the animal developed melena, metrorrhagia, and epistaxis. At gross examination, significant findings included pale tissues, hemothorax, hemoperitoneum, and gallbladder distension with yellow‐green mucoid material, which was submitted for cytology and aerobic culture. Cytologic examination of a smear of gallbladder material showed a highly cellular sample on a proteinaceous background. Most of the cells were degenerate neutrophils, and short rods were identified intracellularly and extracellularly. Case 3 was a 13‐year‐old male AN with a one‐year history of mild anemia and weight loss that was unresponsive to medical intervention. Based on the continued weight loss and poor response to treatment, the animal was humanely euthanized and submitted for necropsy. The peritoneal cavity contained approximately 100 mL of fibrinous effusion. The liver was mildly enlarged (3.65% BW; Table 4), and the gallbladder was distended by opaque tan material, which was sampled for aerobic culture. A section of the jejunum approximately 30 cm from the stomach had a focally extensive intussusception. On histology, the liver had random foci of hepatocellular liquefactive necrosis surrounded by neutrophils. The gallbladder mucosa was hyperplastic, and the submucosa was edematous with a moderate infiltrate of eosinophils, macrophages, and fewer lymphocytes. Case 4 was a 15‐year‐old male AN with a clinical history of weight loss, jaundice, and elevated liver enzymes (Table 3). The animal had a mild normocytic normochromic anemia and decreased platelets. Based on poor prognosis, the animal was euthanized and submitted for necropsy. The gallbladder was distended, yellow to tan, and the wall was thickened up to 3 mm (Figure 1A). The contents of the gallbladder were opaque and tan with flecks of hemorrhage and brown friable material (Figure 1B). Histologically, the gallbladder wall was edematous, and the lumen contained an exudate of degenerate neutrophils, fibrin (Figure 1C), and colonies of short rod‐shaped bacteria (Figure 1C, inset). The morphology of the bacteria seen in section correlated with the culture results of *E. coli*.

**TABLE 3:** Serum chemistry values of liver specific enzymes and metabolites for cases with antemortem bloodwork.

Diagnosis	Case #	Bilirubin [0.1–0.5]	ALT [23–154]	AST [59.0–398]	GGT [0–22.1]	CHOLesterol [44.0–232]	TG [29–232.0]
Cholecystitis	4	**10.25**	**487**	**2709**	**1120**	185	**323**
Cholecystitis	5	**5.62**	59	260	**610**	**274**	**686**
Vitamin E deficiency	6	**0.55**	**1085**	**954**	**34**	78	156
Cardiac‐associated	10	**0.95**	**395**	**695**	**138**	171	**440**
End‐stage	20	**1.49**	**250**	**2427**	**215**	**—**	122
Cholangio‐carcinoma	21	**40.11**	142	**857**	**4220**	**603**	**1499**

*Note:* Bold values indicate elevations outside of the reference interval.

Abbreviations: ALT, alanine aminotransferase; AST, aspartate aminotransferase; GGT, gamma‐glutamyl transferase; TG, triglycerides.

A 17‐year‐old female AA (Case 5) was humanely euthanized after being diagnosed with acute cholangiohepatitis on routine bloodwork collected prior to elective sedation (Table 3). At necropsy, the animal was in poor body condition and had diffuse seborrhea. The liver was moderately enlarged (5.20% BW; Table 4), and the gallbladder was distended. Bile could not be expressed through the common bile duct, and the contents of the gallbladder were tan and mucoid. Histologically, two concurrent processes were noted: (1) chronic hepatocellular degeneration and loss with periportal bridging fibrosis and bile duct hyperplasia and (2) acute neutrophilic cholangiohepatitis with hepatocellular necrosis and reactive fibroplasia, suggesting an ascending infection combined with chronic obstruction of the bile duct.

**TABLE 4:** Signalment data and percent body weight in owl monkeys with liver disease.

Case #	Sex	Age	Terminal body weight (g)	Liver weight (g)	% Body weight
1	M	14	750	39.11	5.21
2	F	9	1100	47.00	4.27
3	M	13	846	30.867	3.65
4	M	15	692	29.08	4.20
5	F	16	773	39.01	5.20
6	F	8	843	36.825	4.91
7	F	1	846	59.38	7.92
8	M	4	692	35.814	4.78
9	M	12	1174	48.6	4.14
10	M	16	999	42.202	4.22
11	M	9	951	53.486	5.62
12	F	20	626	33.08	5.28
13	M	18	910	38.12	4.19
14	M	15	922	53.215	5.77
15	F	10	640	25.507	3.99
16	M	8	847	42.79	5.05
17	M	13	806	34.81	4.32
18	M	23	563	21.86	3.88
19	M	15	829	30.11	3.63
20	F	13	770	43.85	5.69
21	M	12	960	91.643	9.55
22	F	18	1095	126.31	11.54

Abbreviation: g, grams.

### 3.2 Acute Centrilobular Necrosis

Three AG (Cases 6–8; Table 4) with a median age of 6 years were evaluated clinically for lethargy and pallor. Only one animal (Case 6; 8‐year‐old female) had a complete blood count performed prior to euthanasia, which revealed a hematocrit of 5.8% [reference interval 39.6–64.5], consistent with severe anemia. Case 6 also had serum chemistry results available (Table 3), which indicated elevations in GGT, ALT, and AST suggestive of hepatobiliary disease and intravascular hemolysis. The animals failed to respond to supportive therapy and were euthanized. At necropsy, the livers had an enhanced reticular pattern, which correlated with acute centrilobular coagulative necrosis from anemia induced hypoxia. The sinusoids were expanded by nucleated erythrocytes and rubricytes. Additional gross lesions included thin watery blood, multifocal areas of acute hemorrhage in the cerebral cortex (cerebral ring hemorrhage), pale organs, and in one case, melena.

### 3.3 Cardiac‐Associated Hepatopathy

**FIGURE 2:**
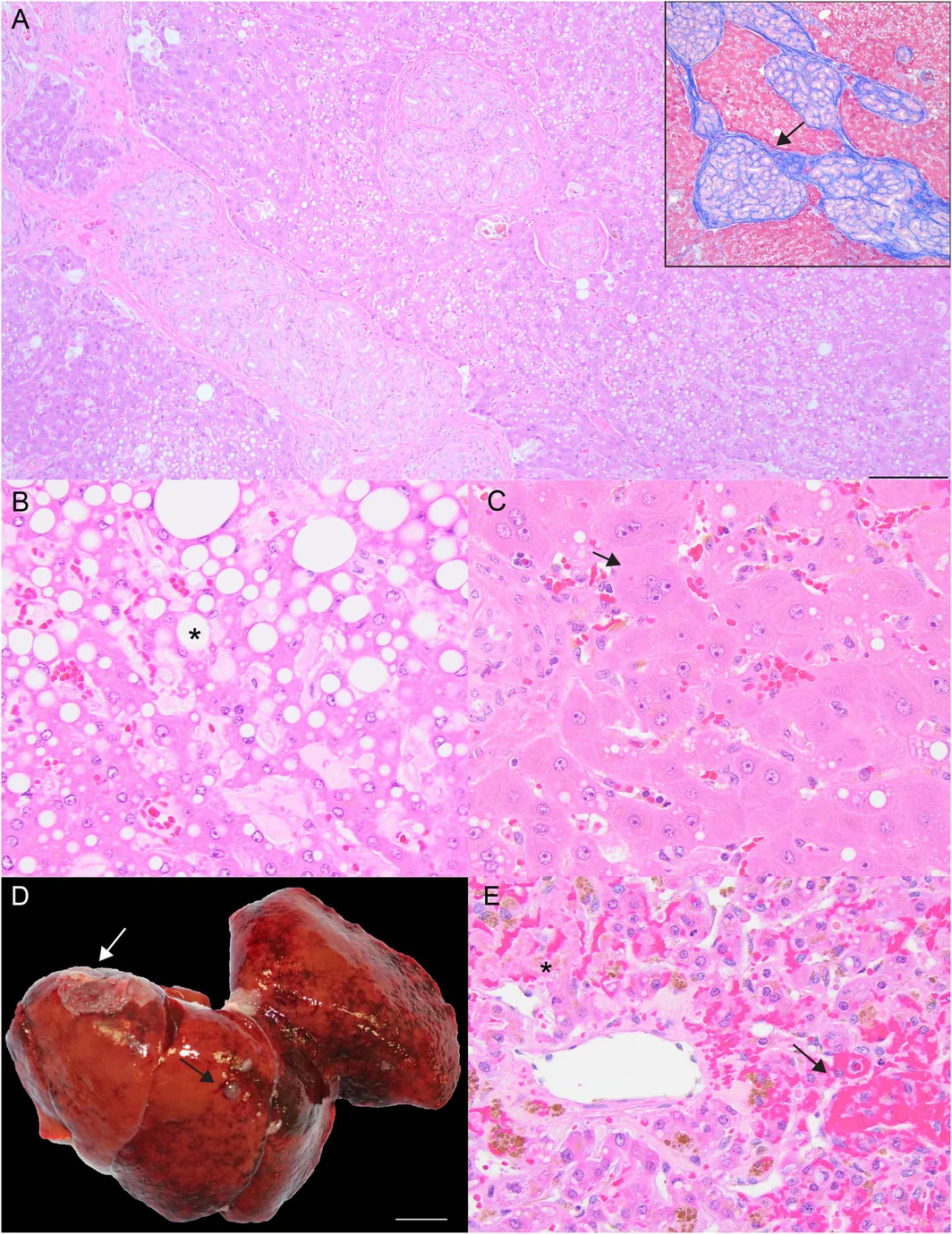
Histopathologic findings of cardiac‐associated liver lesions in *Aotus* spp. (A) Case 9, Male *A. nancymai* with chronic hepatopathy secondary to dilated cardiomyopathy and heart failure. Histologic features included periportal bridging fibrosis embedded with numerous small bile duct profiles, hepatocellular cytoplasmic lipid vacuolation. Inset, Dissecting bands of mature fibrous connective tissue (arrow) with embedded small bile duct profiles. Masson's trichrome, 2×. (B) Case 12, *A. nancymai* demonstrating hepatocyte cytoplasmic lipid vacuolation (*). HE, 20×. (C) Case 12, *A. nancymai* with hepatocellular regeneration characterized by hepatocellular karyomegaly and polyploidy (arrow). HE, 20×. (D) Case 10, male *A. nancymai* with bilateral congestive heart failure. The liver is enlarged with an enhanced reticular pattern. Plaques of fibrin are adhered to the capsular surface (white arrow). Scattered throughout the liver are biliary cysts (black arrow) and depressed dark red foci interpreted as peliosis hepatis. Scale bar = 1 cm. (E) Case 10, Centrilobular hepatocytes have acute coagulative necrosis (*) and the sinusoids are expanded by red blood cells (arrow) consistent with peliosis hepatis. HE, 20×.

Cases 9–14 had a median age of 15.5 years and had been medically managed for cardiomyopathy. Case 9 cardiac function was evaluated by ultrasound, which revealed dilated cardiomyopathy with poor contractility. In each case, clinical endpoints were reached, and the decision was made to humanely euthanize the animals. Gross examination for the six cases revealed similar lesions: dilated or hypertrophic cardiomyopathy, tricavitary transudate to fibrinous effusions, enlarged reactive mediastinal lymph nodes, brown‐tinged lungs consistent with chronic pulmonary hemorrhage, and an enhanced reticular pattern in the liver suggestive of chronic passive congestion. The predominant histologic feature was periportal and centrilobular bridging fibrosis with mature connective tissue embedded with numerous small bile duct profiles lined by low cuboidal epithelium (Figure 2A). Masson's trichrome was applied to highlight the dissecting bands of mature fibrosis between portal and centrilobular areas (Figure 2A, inset). The bands of fibrosis created islands of regenerative hepatocytes with lipid‐type cytoplasmic vacuolation (Figure 2B). Regenerating hepatocytes had frequent karyomegaly and polyploidy (Figure 2C). In acute cases of portal hypertension and hypoxia, centrilobular hepatocellular coagulative necrosis with hemorrhage and sinusoidal dilation was the predominant lesion. In more chronically affected animals, the liver had multifocal raised 1–2 cm nodules that on cut section were pale and slightly firm interpreted as regenerative nodules. These nodules had originally been diagnosed as neoplastic, but reticulin staining confirmed that the hepatocellular trabeculae were minimally thickened up to 2–3 cell layers consistent with regenerative hyperplasia.

Case 10 was a 16‐year‐old male AN with serum chemistry evaluated (Table 3), which demonstrated increased liver enzyme activity and total bilirubin that suggested concurrent biliary tree disease. On gross exam, the liver was enlarged (4.22% BW; Table 4) and diffusely orange tinged with multifocal 0.5–1 cm, soft raised fluid filled cysts interpreted as biliary cysts and multifocal dark red to black depressed areas, predominantly in the subcapsular parenchyma (Figure 2D). Additionally, plaques of fibrin were loosely adhered to the capsular surface between the liver and diaphragm and individual liver lobes (Figure 2D). The gallbladder was small, and the common bile duct wall appeared irregularly thickened up to 1.5 mm. Histologically, the liver had centrilobular hepatocellular coagulative necrosis attributed to hypoxia or portal hypertension (Figure 2E). Additionally, multifocal areas of the sinusoids were dilated and contained erythrocytes interpreted as the phlebatic form of peliosis hepatis (Figure 2E), and the hepatic parenchyma was multifocally replaced by variably sized cysts lined with low cuboidal epithelium interpreted as biliary cysts. Increased total bilirubin and GGT were likely secondary to lesions in the common bile duct, which had mural fibrosis and medial hypertrophy and hyalinization of the submucosal vessels and reactive plump endothelial cells that narrowed the lumen. Vascular lesions in the small muscular arteries and arterioles suggest systemic and portal hypertension contributed to the pathogenesis of the hepatic lesions. Similar arteriosclerosis lesions were identified in vessels of the heart and kidneys.

### 3.4 End‐Stage Liver Disease of Undetermined Etiology

One 10‐year‐old female AV (Case 15) was diagnosed with chronic hepatopathy with an undetermined inciting cause. Clinical signs were vague and included weight loss and lethargy. At necropsy, the animal was thin with minimal internal fat stores. The liver was mildly enlarged (3.98% BW; Table 4) with rounded margins and a cobblestone appearance to the parenchyma. The predominant histologic feature was periportal and centrilobular bridging fibrosis with mature connective tissue embedded with numerous small bile duct profiles lined by low cuboidal epithelium. Co‐morbidities diagnosed at post‐mortem examination included lymphoplasmacytic enterotyphlitis, serous atrophy of fat, and atrophy of exocrine pancreatic acini. All other tissues examined, including the heart and kidneys, were unremarkable, and the cause for end‐stage liver disease was not apparent.

### 3.5 Hemosiderosis

**FIGURE 3:**
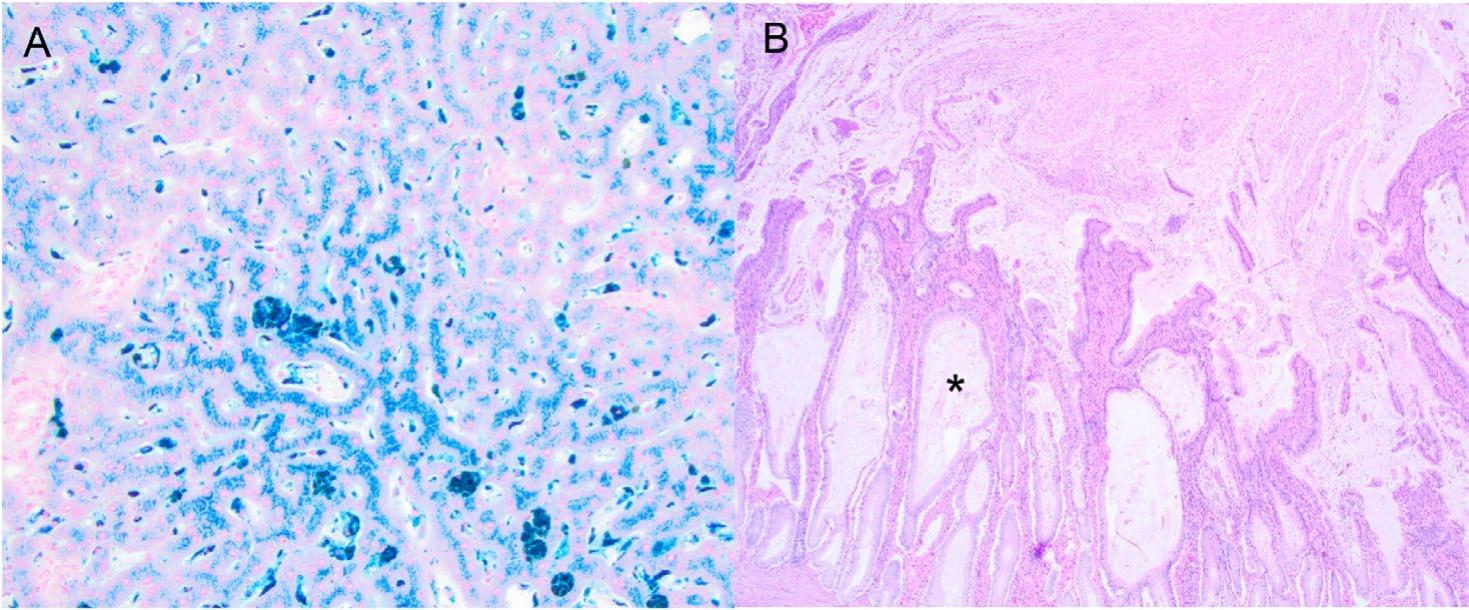
Incidental lesions in the livers of *Aotus* spp. (A) Case 13, *A. nancymai* with chronic hemosiderosis leading to iron (arrow) accumulation in hepatocytes and Kupffer cells. 4×, Perls' iron stain, Neutral red counterstain. (B) Case 16, *A. nancymai* with cystic mucinous hyperplasia of the gallbladder mucosa. Note the dilated glands (*) containing eosinophilic mucinous material. HE, 2×.

Cases 15–18 represented cases of a common background lesion in the colony. The cases included four adult male owl monkeys (2 AG; 2 AN) with a median age of 14 years. No gross lesions were noted in the livers. The histologic lesion was diffuse moderate to severe brown granular to globular cytoplasmic pigment accumulation in hepatocytes and Kupffer cells. Perls' stain confirmed that the intracytoplasmic pigment was iron (Figure 3A). This lesion is considered an incidental finding because hepatocellular intracytoplasmic iron was not accompanied by hepatocellular degeneration or necrosis. In the affected AG, the animals also had extensive deposition of iron in histiocytes of the bone marrow, lung, glomeruli, and spleen. Accumulation of iron does not appear to be toxic to the hepatocytes as the change was not accompanied by elevations in liver enzymes and systemic iron levels were within normal limits for the cases with bloodwork available.

### 3.6 Cystic Mucinous Hyperplasia

A 13‐year‐old female AN (Case 19) died spontaneously. At necropsy, the liver was moderately enlarged (5.69% BW; Table 4) with an enhanced reticular pattern, and the gallbladder mucosa was thickened up to 2 mm. Histologically, the gallbladder mucosa was diffusely hyperplastic with multifocal mucus‐filled cysts, which is consistent with cystic mucinous hyperplasia (Figure 3B). The lumen of the gallbladder contained mucus and degenerate neutrophils and karyorrhectic debris, which suggests inflammation as the underlying cause for cystic hyperplasia. Additional diagnoses included moderate to severe cardiac interstitial fibrosis, moderate nephrosclerosis and glomerulosclerosis, and arteriosclerosis indicating hypertension.

### 3.7 Neoplasia

Two cases of intrahepatic cholangiocarcinoma were diagnosed in an 18‐year‐old female AN and a 12‐year‐old male AN (Cases 21–22, respectively). The clinical signs for Case 21 included icterus, lethargy, ascites, and hepatomegaly; serum chemistry collected 2 weeks before necropsy revealed elevated liver enzymes, triglycerides, cholesterol, and marked hyperbilirubinemia (Table 3). Case 22 was found deceased with no premonitory signs.

**FIGURE 4:**
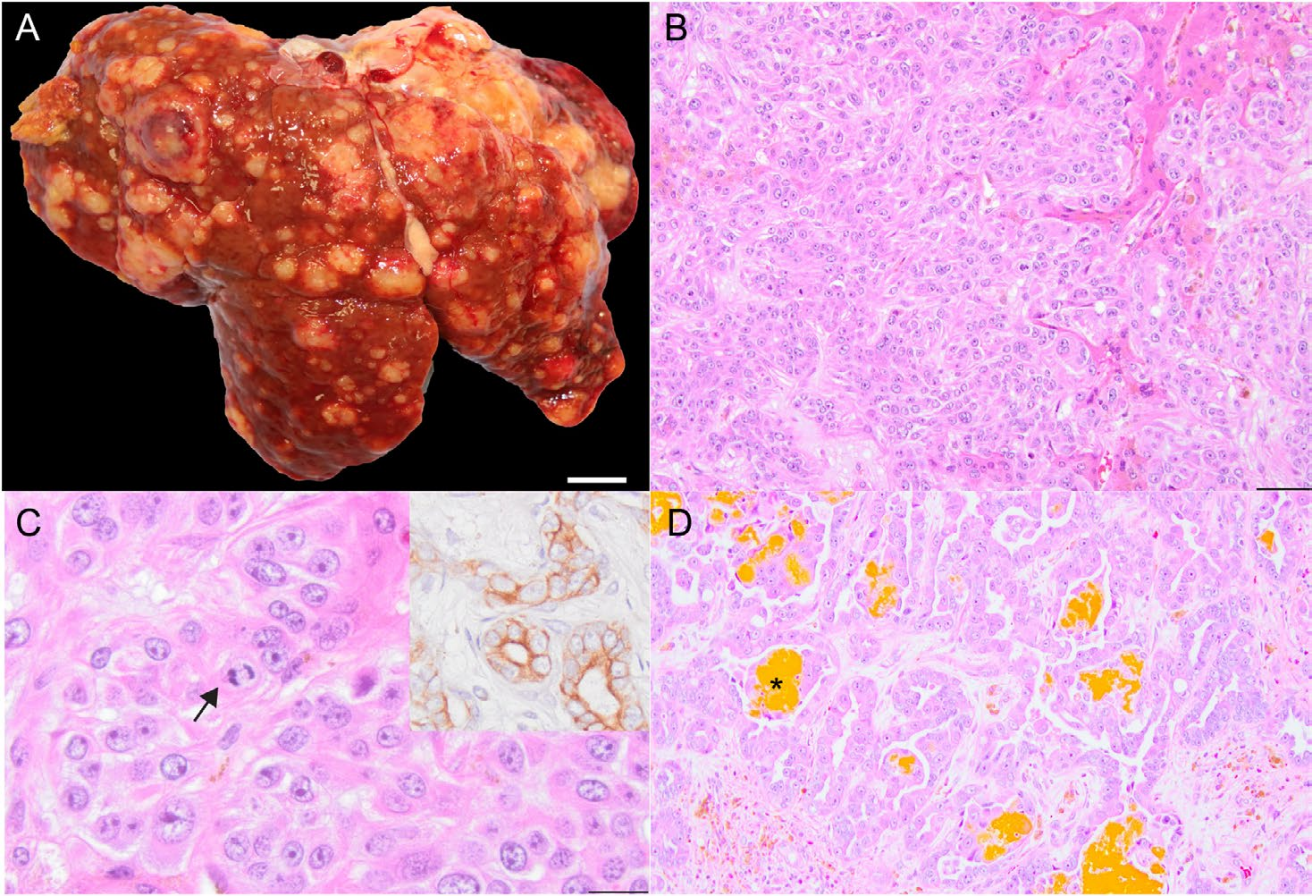
Intrahepatic cholangiocarcinoma in Case 22 with a markedly enlarged liver. (A) Case 22, male *A. nancymai* . Scattered throughout all liver lobes are numerous tan umbilicated nodules. Scale bar = 1 cm. (B) Neoplastic cells dissect between and replace hepatocytes. Cells are arranged in nests on a moderate fibrovascular stroma. HE, 10×. (C) Neoplastic cholangiocytes are cuboidal with moderate amounts of eosinophilic cytoplasm and a round nucleus with finely stippled chromatin and a single round nucleolus. The cells have moderate anisocytosis and anisokaryosis. Mitotic figures are frequent (arrow). Inset, Neoplastic cells display strong cytoplasmic staining with cytokeratin‐7, diaminobenzidine with hematoxylin counterstain, 40×. (D) Neoplastic ducts contained bright yellow pigment (*) interpreted as bile. HE, 10×.

Case 21 had a markedly enlarged liver (9.46% BW; Table 4) with multifocal, variably sized cystic nodules that contained thick opaque, green‐tinged material. In Case 22, variably sized umbilicated firm nodules (Figure 4A) were scattered throughout the enlarged liver (11.535% BW; Table 4) with extensive peritoneal lymph node metastasis, carcinomatosis, and distant embolic metastasis to the lung. The histologic appearance of the intrahepatic cholangiocarcinomas was similar in both cases. Approximately 60% of the hepatic parenchyma was effaced and replaced by a neoplastic population that formed nests and tubules lined by one to several layers of cuboidal epithelial cells (Figure 4B). Neoplastic cells had moderate amounts of eosinophilic cytoplasm and a round basally located nucleus with finely stippled chromatin and 1 round nucleolus (Figure 4C). Immunohistochemistry for cytokeratin 7, a marker of bile duct epithelium, demonstrated diffuse membranous and cytoplasmic staining of the neoplastic population (Figure 4B, inset). Cells rarely had an apical brush border of microvilli. Neoplastic cells were supported by an abundant desmoplastic stroma with scattered hemosiderin‐laden macrophages embedded. Bile ducts were often dilated and contained bright yellow material interpreted as bile and karyorrhectic debris, foamy macrophages, and degenerate neutrophils (Figure 4D). Remaining hepatocytes were isolated in small cords and nodules separated by mature fibrous connective tissue with small non‐neoplastic bile duct profiles interpreted as biliary hyperplasia.

## 4 Discussion

Hepatobiliary disease was diagnosed in 6.96% of the population based on a combination of changes in liver enzymes on serum chemistry and gross and histologic lesions. The most consistent clinical sign across the cases was weight loss and lethargy. Interestingly, icterus was not a defining feature of liver disease despite elevations in bilirubin detected in the serum; due to the pigmented skin and thick haircoat, mild icterus can be difficult to detect without close examination of the sclera or mucous membranes. Hyperbilirubinemia and elevated enzymes such as GGT, AST, and ALT were consistently noted regardless of the final diagnosis. However, hyperbilirubinemia was most pronounced in Case 21, which also had evidence of bile casts. Icterus was confirmed at necropsy in Cases 4, 17, and 21 that had the most dramatic increases in bilirubin. Changes in GGT, AST, ALT, and cholesterol are suggestive of liver disease but can also be elevated in heart and kidney disease, which are common comorbidities in owl monkeys [7, 15, 16]. Specifically, elevations in AST can occur with muscle injury from acute myocardial infarction or active myocardial degeneration [17]. Takeshita et al. evaluated the ultrasonographic appearance of the liver in *A. azarae infulatus* and correlated findings with liver enzyme values, reporting evidence of hepatic pathology in 25% of animals. Among individuals with ultrasonographic liver abnormalities, liver enzyme elevations showed poor correlation with imaging findings. Direct comparison between that study and the present investigation is limited because the current study is retrospective, details findings in an older population, and many animals lacked complete serum chemistry data.

Hepatic disease attributed to infectious etiologies was diagnosed in 21.7% of the cases reviewed. The pathogenesis was primarily considered an ascending infection from reflux of the intestinal contents, but it could also be associated with choleliths [18]. Cases 3 and 4 had friable material admixed with fibrin in the gallbladder contents that could represent cholelith material. Cholecystitis is uncommonly reported in research populations of nonhuman primates but has been associated with Gram‐negative bacterial infections, protozoa, or trematode infestation [10, 18, 19, 20, 21, 22]. *E. coli* was the most common bacteria isolated by culture, which is consistent with cases in domestic species [18, 23, 24]. In common marmosets ( *Callithrix jacchus* ), *Pseudomonas aeruginosa* was the most common isolate in animals with cholecystitis [19, 25]. Immune suppressed macaques (*Macaca* spp.) may exhibit proliferative cholecystitis secondary to parasitic infection with *Cryptosporidium* spp. or *Enterocytozoon bieneusi* [10]. Common marmosets ( *C. jacchus* ) have also been diagnosed with proliferative cholecystitis secondary to trematode infestation with *Platynosomum* sp. [20, 21].

The most common diagnosis in this study population was cardiac‐associated liver disease. The gross lesions associated with chronic passive congestion from heart disease are nonspecific but include hepatomegaly and an enhanced reticular pattern. These morphologies were noted in all cases with cardiac‐associated liver disease. Centrilobular hypoxia and coagulative necrosis of the hepatocytes result from increased hydrostatic pressure in the portal venous system and are a well‐characterized sequelae of heart failure [18]. Portal hypertension can lead to bridging fibrosis between portal and centrilobular areas, which further exacerbates fibrosis. Cardiomyopathy is often seen in aged *Aotus* spp., and the clinical syndrome has been extensively characterized [15, 16, 26, 27, 28, 29, 30, 31]. Several animals with cardiac‐associated heart disease had lesions consistent with end‐stage hepatopathy or cirrhosis based on the formation of regenerative nodules and bridging fibrosis that involved portal areas and central veins [18].

Regenerative nodules are not often documented in veterinary species other than dogs as a part of chronic hepatopathy, but in the owl monkey cases described here, the constellation of gross and histologic evidence supports the diagnosis [18]. Nodular hyperplasia has been described in 2 cynomolgus macaques ( *Macaca fascicularis* ), but the lesion was noted in young animals (< 3‐years‐old) and was not associated with further evidence of hepatocellular degeneration and fibrosis [32]. Cirrhosis is often a diagnosis in aged animals and is less commonly reported in younger animals unless incited by chronic exposure to a toxin or infectious etiology [18]. Perhaps due to the young age of the research population, this lesion has not yet been described in *Aotus* spp.; however, as the breeding population ages this type of lesion will likely become more clinically relevant as a cause of morbidity and health and welfare concerns.

In addition to lesions associated with chronic cardiomyopathy, Case 10 had microscopic evidence of cholelith material in the gallbladder lumen that may have caused GGT elevations detected on serum chemistry. The most common type of cholelith described in owl monkeys is cholesterol choleliths, which are reportedly not associated with histologic lesions in the gallbladder mucosa [11]. Cholesterol choleliths have been reported in the owl monkeys and can be experimentally induced by increased dietary fat for 5 weeks [11, 33, 34]. It is possible that the case diagnosed with idiopathic end‐stage hepatopathy had cholelithiasis that was not detected at the time of necropsy.

Vitamin E deficiency in owl monkeys is considered karyotype specific with karyotypes II, III, and IV considered most susceptible [35]. Chromosomal analysis of Colombian *Aotus* species indicates that karyotypes II, III, and IV are members of *Aotus griseimembra* [36, 37]. Three AG developed lesions associated with vitamin E deficiency including anemia and centrilobular hepatocellular necrosis with intrasinusoidal nucleated erythrocytes after decreased frequency of supplemental vitamin E administration. Based on the findings, the previous vitamin E schedule was resumed, and no new cases of vitamin E deficiency occurred. The lesions are consistent with those reported in the literature, but the diagnosis is presumptive since vitamin E levels were not evaluated in serum or tissue [12, 38, 39, 40, 41]. No other species of owl monkey housed at the center developed lesions associated with vitamin E deficiency during the period of decreased frequency of administration, which supports the enhanced requirements of *A. griseimembra* for supplemental non‐dietary vitamin E.

Hemosiderosis is a common finding in all species of *Aotus* housed at the KCCMR, and the results reported herein are a representative rather than exhaustive list. Similar to the report by de Souza et al., hemosiderosis is considered the appropriate diagnosis for these cases described herein because the accumulation of iron in the hepatocytes and Kupffer cells was not accompanied by hepatocellular degeneration/necrosis, inflammation, or fibrosis as has been reported for hemochromatosis in humans [5, 42]. Interestingly, total iron concentration in serum chemistry was not elevated in any of these cases suggesting a disconnect between iron deposition in the liver and the ability of clinical tests to detect it. Enhanced storage of iron has been reported in numerous lemur species, great apes, and nonhuman primates of research interest [5, 43, 44, 45, 46, 47, 48]. Lemurs and common marmosets have a known predilection for increased iron storage in hepatocytes, and the pathogenesis is presumptively due to enhanced enterocyte uptake combined with excess iron in the diet compared to requirements [45, 47, 49, 50]. The role between dietary iron and hemosiderosis was investigated in common marmosets ( *Callithrix jacchus* ), which found that a high iron diet led to increased mortality compared to marmosets fed a low iron diet. Despite increased mortality from a high iron diet, bloodwork parameters such as serum iron concentrations, hematocrit, or total iron binding capacity did not demonstrate statistically significant differences between groups and were not correlated with hepatic iron [49]. However, a later study in a zoo population of callithridae identified a correlation between ferritin and hepatic iron, which suggests ferritin may be a more useful noninvasive indicator of hepatic iron levels [50]. Similar to the owl monkeys, increased iron pigment in hepatocytes was not accompanied by hepatocellular death or degeneration in common marmosets [49].

A single case of cystic mucinous hyperplasia was diagnosed in a 13‐year‐old *A. nancymai* . This condition is reported in dogs and rarely in cats and is considered an incidental finding in those species [18]. To the authors' knowledge, this is the first case documented in a nonhuman primate species. The lesion was accompanied by neutrophilic cholangiohepatitis, so it is possible mucinous hyperplasia was a response to inflammation.

Neoplasia is an uncommon diagnosis in owl monkeys, but a single case report described a gallbladder adenocarcinoma with regional metastasis to the draining lymph node [9]. Spontaneous cholangiocarcinoma has been reported in a variety of nonhuman primate species and can be induced as an experimental model in rhesus macaques ( *M. mulatta* ) [51, 52, 53, 54]. The cases described herein had intrahepatic cholangiocarcinoma with a characteristic gross appearance of umbilicated nodules scattered throughout the liver. One case had extensive metastasis with carcinomatosis of the peritoneal cavity and metastasis to the regional lymph nodes and lung. As the research breeding population advances in age, more cases of neoplasia could be reported. In humans, cholangiocarcinoma is often idiopathic but can be associated with trematode infestation in endemic regions, cholelithiasis, sclerosing cholangitis, cholecystitis, or genetic predisposition [55]. The owl monkeys at the KCCMR are part of a closed population housed indoors and have no history of trematode parasitism. Chronic cholecystitis has been reported in owl monkeys, but a correlation with cholangiocarcinoma remains to be determined.

## 5 Conclusions

The spectrum of hepatobiliary pathology in owl monkeys is broader than previous reports have suggested. This report highlights previously undescribed conditions in *Aotus* spp. including infectious cholecystitis and cholangiocarcinoma and provides a baseline reference for evaluating liver disease in the species by correlating serum chemistry changes with macroscopic pathology.

## Conflicts of Interest

The authors declare no conflicts of interest.

## Data Availability

The data that support the findings of this study are available from the corresponding author upon reasonable request.
